# The Gut Microbiome as a Major Regulator of the Gut-Skin Axis

**DOI:** 10.3389/fmicb.2018.01459

**Published:** 2018-07-10

**Authors:** Iman Salem, Amy Ramser, Nancy Isham, Mahmoud A. Ghannoum

**Affiliations:** ^1^Center for Medical Mycology, Department of Dermatology, Case Western Reserve University, Cleveland, OH, United States; ^2^Dermatology, University Hospitals Cleveland Medical Center, Cleveland, OH, United States

**Keywords:** gut microbiome, skin homeostasis, acne vulgaris, atopic dermatitis, psoriasis, probiotics

## Abstract

The adult intestine hosts a myriad of diverse bacterial species that reside mostly in the lower gut maintaining a symbiosis with the human habitat. In the current review, we describe the neoteric advancement in our comprehension of how the gut microbiota communicates with the skin as one of the main regulators in the gut-skin axis. We attempted to explore how this potential link affects skin differentiation and keratinization, its influence on modulating the cutaneous immune response in various diseases, and finally how to take advantage of this communication in the control of different skin conditions.

## Introduction

**FIGURE 1:**
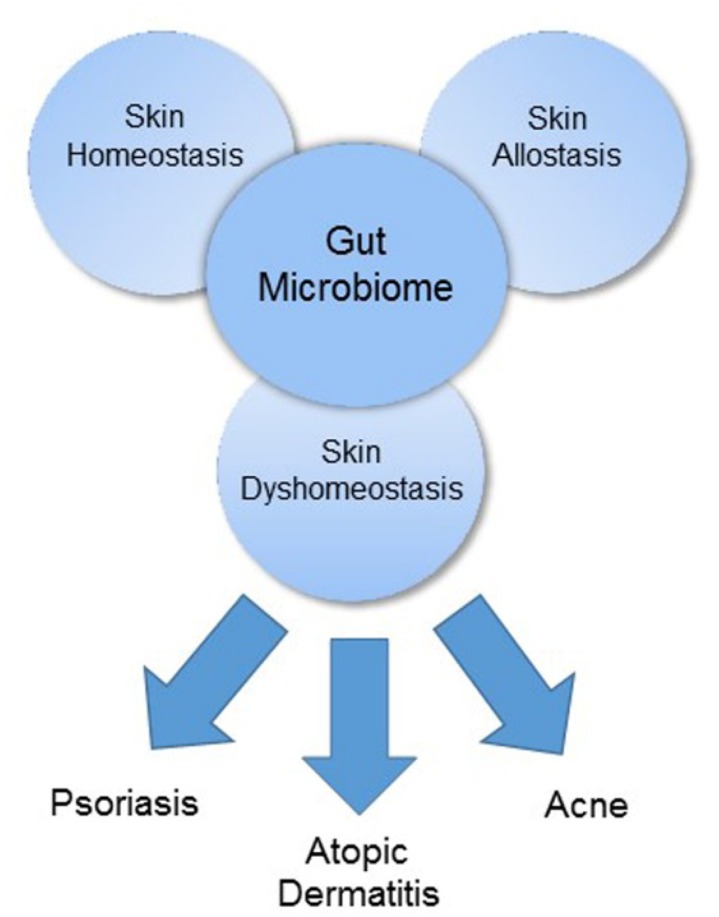
The gut microbiome as a major regulator of the gut-skin axis.

The gut and skin, densely vascularized and richly innervated organs with crucial immune and neuroendocrine roles, are uniquely related in purpose and function (O’Neill et al., 2016). As our primary interface with the external environment, both organs are essential to the maintenance of physiologic homeostasis. Cumulative evidence has demonstrated an intimate, bidirectional connection between the gut and skin, and numerous studies link gastrointestinal (GI) health to skin homeostasis and allostasis (**Figure 1**) (Levkovich et al., 2013; O’Neill et al., 2016). GI disorders are often accompanied by cutaneous manifestations and the GI system, particularly the gut microbiome, appears to participate in the pathophysiology of many inflammatory disorders (Shah et al., 2013; Thrash et al., 2013; Gloster et al., 2016). In this review, we will discuss the gut microbiome’s contribution to three common skin disorders – acne, atopic dermatitis (AD), and psoriasis – (**Figure 1**) as well as review data on how the microbiome’s influence can be harnessed for therapeutic purpose via probiotic supplementation.

## Gut Microbial Ecology

Our gut microbiome is a vast collection of bacteria, viruses, fungi, and protozoa colonizing our GI system (Ipci et al., 2017). This collection of microbes outnumbers host cells 10-fold and contains genetic material 150 times greater than the host’s own karyosome (Wu and Lewis, 2013; Ipci et al., 2017). Recent advances in metagenomics and the advent of high-throughput DNA-sequencing technology has enhanced our understanding of the microbiome and its dynamic influence on human health and pathology (Boyle et al., 2011; Moore-Connors et al., 2016).

The intestinal microbiome provides important metabolic and immune benefits to the host. Gut flora contribute to the breakdown of indigestible complex polysaccharides and are vital to the production of certain nutritional components such as vitamin K. The gut microbiome’s influence on the host immune system is vast, and the relationship is intricately regulated to both enable immune tolerance of dietary and environmental antigens and provide protection against potential pathogens. The intestinal microbiome protects against invasion by exogenous pathogens directly, by competitively binding to epithelial cells, and indirectly, by triggering immunoprotective responses (Boyle et al., 2011; Kosiewicz et al., 2014).

Commensal bacteria prime the gut immune system through specific interactions between bacterial antigens and pattern recognition receptors expressed by a variety of host cells (Kosiewicz et al., 2014). For example, gut microbes are a source of peptidoglycan capable of altering the expression of toll-like receptors (TLRs), pattern recognition receptors on the surface of many innate immune cells. TLRs recognize pathogen associated molecular patterns and trigger cascades of events linking the innate to the adaptive immune system through the activation of the nuclear factor kappa B (NF-κB) signaling pathway (Macpherson and Harris, 2004; Clarke et al., 2010; Thomas and Versalovic, 2010; Boyle et al., 2011; Kosiewicz et al., 2014). The contribution of the gut microbiome to the adaptive immune system has been well-characterized and involves the induction of immunoglobulin A and the maintenance of homeostasis between effector T cells (Th1, Th2, and Th17) and regulatory T cells (Kosiewicz et al., 2014). Certain microbes can also contribute to intestinal epithelial barrier function via “cross-talk” with elements of mucosal immunity (Bik, 2009; Purchiaroni et al., 2013; Kosiewicz et al., 2014; Rajilić-Stojanović and de Vos, 2014; Sirisinha, 2016). For instance, one commensal gut microbe, *Lactobacillus* LGG, secretes p40, a protein capable of suppressing cytokine-mediated apoptosis and epithelial barrier disruption. Another species, *Escherichia coli* Nissle, contains flagella capable of inducing β-defensin 2 in epithelial cells (Kosiewicz et al., 2014).

Many human and animal studies suggest that the intestinal microbiome’s influence extends beyond the gut, and in fact contributes to the function, and dysfunction, of distant organ systems (Levkovich et al., 2013; Kim Y.G. et al., 2014). Short chain fatty acids (SCFAs), products of dietary fibers fermented by components of the gut microbiome, demonstrate a protective role against the development of inflammatory disorders including arthritis and allergy, in addition to colitis (Kim Y.G. et al., 2014). Intestinal dysbiosis, in the form of unbalanced bacterial composition or aberrant immune reactions to commensal flora, has been linked to metabolic, neurodegenerative, and neoplastic diseases. Altered gut flora may favor the production of effector over regulatory T cells, thereby contributing to the development of autoimmune disorders. For example, segmented filamentous bacteria in the gut have been associated with a variety of Th17-mediated diseases. Through mechanisms not yet completely understood, the gut microbiome’s influence clearly extends beyond the GI system. One distant organ known to have a particularly complex connection with the gut is the skin (Levkovich et al., 2013; Kim Y.G. et al., 2014; Kosiewicz et al., 2014).

## Role of the Gut Microbiome in Skin Homeostasis

The skin effectively performs its functions – protection, temperature regulation, water retention, and more – when in a state of homeostasis. As an organ undergoing constant renewal, effective epidermal turnover, the process by which the skin regenerates itself, is essential to maintaining this state. Epidermal cells originate from stem cells in the basal layer of the epidermis and then undergo morphologic change while migrating to the skin surface. Cells differentiate into three cell types – basal cells, spinous cells, and granule cells – before ultimately becoming the corneocytes that make up the outermost layer of the epidermis, the stratum corneum. This process of epidermal differentiation, also referred to as keratinization, is under the control of dedicated transcriptional programs. For example, the expression of *KRT5/K5* and *KRT14*, the genes which encode keratin 5 and keratin 14, respectively, is downregulated as migrating cells move outward, while the expression of genes encoding *KRT1* and *KRT10* is upregulated (Baba et al., 2012; Weaver et al., 2013; Gaur et al., 2014; Abhishek et al., 2016). Ultimately, this highly regulated process results in a stratum corneum consisting of approximately 15 layers of densely keratinized, stratified, and anucleated corneocytes held together with multiple lipid bilayers in a “brick and mortar” model. The corneocytes serve as the bricks, while ceramides, cholesterol, fatty acids, and cholesterol esters make up the mortar that holds the bricks together. When epidermal turnover functions appropriately, the resulting brick and mortar structure serves as an effective skin barrier with the ability to limit evaporation, preserve skin moisture, and protect from invasion by foreign organisms and substances (Baba et al., 2012; Weaver et al., 2013; Gaur et al., 2014). Through its influence on the signaling pathways that coordinate this process essential to skin homeostasis, the gut microbiome impacts integumentary health (O’Neill et al., 2016).

Though not yet fully explored, the mechanisms by which intestinal microbiota exert their influence on skin homeostasis appear to be related to the modulatory effect of gut commensals on systemic immunity (O’Neill et al., 2016). Certain gut microbes and metabolites – retinoic acid, polysaccharide A from *Bacteroides fragilis, Faecalibacterium prausnitzii*, and bacteria belonging to *Clostridium* cluster IV and XI promote the accumulation of regulatory T cells, lymphocytes which facilitate anti-inflammatory responses (Forbes et al., 2015). Segmented filamentous bacteria, alternatively, promote the accumulation of pro-inflammatory Th17 and Th1 cells. SCFAs, particularly butyrate, suppress immune responses by inhibiting inflammatory cells’ proliferation, migration, adhesion, and cytokine production. In addition, through their inhibition of histone deacetylase and inactivation of NF-κB signaling pathways, SCFAs regulate both the activation and apoptosis of immune cells. The inhibition of histone deacetylase promotes the proliferation of regulatory cells involved in various cutaneous physiologic functions including regulation of hair follicle stem cell differentiation and wound healing (Meijer et al., 2010; Loser and Beissert, 2012; Samuelson et al., 2015). In addition, there is new evidence that the intestinal microbiome may impact cutaneous physiology, pathology, and immune response more directly, through the metastasis of gut microbiota and their metabolites to the skin (Samuelson et al., 2015; O’Neill et al., 2016). In cases of disturbed intestinal barriers, intestinal bacteria as well as intestinal microbiota metabolites have been reported to gain access to the bloodstream, accumulate in the skin, and disrupt skin homeostasis (O’Neill et al., 2016). DNA of intestinal bacteria has been successfully isolated from the plasma of psoriatic patients (O’Neill et al., 2016). These findings represent evidence of a more direct link between the gut microbiome and cutaneous homeostasis that has just begun to be explored.

The gut microbiome appears to influence the skin microbiome as well. SCFAs resulting from fiber fermentation in the gut – propionate, acetate, and butyrate – are believed to play a pivotal role in determining the predominance of certain skin microbiomic profiles which subsequently influence cutaneous immune defense mechanisms. *Propionibacterium*, for example, is a genus capable of producing SCFAs, predominantly acetate and propionic acid. Propionic acid can exhibit a profound antimicrobial effect against USA300, the most prevailing community-acquired methicillin-resistant Staphylococcus aureus (Shu et al., 2013; Samuelson et al., 2015; Schwarz et al., 2017). *S. epidermidis* and *P. acnes* are examples of cutaneous commensals known to tolerate wider SCFA shifts than other flora. Altogether, these findings provide supportive evidence for a functional interactive mechanism between gut and skin.

The beneficial effects of gut bacteria on skin health and appearance have been documented in several rodent and human studies (**Tables 1**, **2**). In a study by Levkovich et al. (2013), mice who received *Lactobacillus reuteri* supplementation experienced increased dermal thickness, enhanced folliculogenesis, and increased sebocyte production which manifested as thicker, shinier fur. In another rodent study, Horii et al. (2014) reported that oral supplementation of *Lactobacillus brevis* SBC8803 in rats resulted in decreased cutaneous arterial sympathetic nerve tone and increased cutaneous blood flow, possibly via increased serotonin release from intestinal enterochromaffin cells and subsequent activation of parasympathetic pathways. A significant decrease in transepidermal water loss (TEWL), a marker of skin barrier function, was noted as well (Horii et al., 2014). This effect was reproduced in human clinical research. After taking *L. brevis* SBC8803 oral supplements for 12 weeks, human subjects had significantly decreased TEWL and significantly increased corneal hydration (Ogawa et al., 2016). In a separate placebo-controlled human study, bacterial supplementation was shown to have a positive effect on skin barrier function (Guéniche et al., 2013). Volunteers who took *Lactobacillus paracasei* NCC2461 supplements for 2 months had decreased skin sensitivity and TEWL, an effect attributed to an observed increase in circulating transforming growth factor beta (TGF-β), a cytokine known to have a favorable effect on barrier integrity (Guéniche et al., 2013; O’Neill et al., 2016). A study by Baba et al. (2006) found that when *Lactobacillus helveticus*-fermented milk whey was introduced to human epidermal keratinocyte cultures, there was increased expression of keratin 10 and involucrin, markers of early and late differentiation, respectively, suggesting that *L. helveticus* can promote epidermal differentiation. In addition, there was a dose-dependent increase in profilaggrin, a protein involved in the terminal differentiation of keratinocytes. Profilaggrin is ultimately cleaved to form filaggrin (FLG), a protein essential to normal epidermal flexibility and hydration, suggesting a potential moisturizing benefit of this bacteria as well (Baba et al., 2006).

**Table 1:** Evidence of beneficial probiotic interventions: animal studies.

Author	Population	Oral probiotic	Clinical response	Proposed mechanism
Chapat et al., 2004	C57BL/6 female mice, MHC classII-deficient (Aβ^∘/∘^) mice	*L. casei* ^∗^DN-114 001 (200μl fermented milk/d with 2 × 10^8^CFU^∗^/d × 26 days)	50% inhibition of contact hypersensitivity response to 2,4-DNFB^∗^	↓ Hapten-specific CD8+ T cell proliferation
Guéniche et al., 2006	Hairless Skh:hr1 mice	*L. johnsonii* (La1) (1×10^8^ CFU/d × 10 days)	Protected against UVR-induced contact hypersensitivity	↓ Epidermal LCs^∗^ density and ↑ IL-10 plasma levels
Baba et al., 2010	Hos:HR-1 hairless mice	*L. helveticus*-fermented milk whey (in distilled water *ad libitum* × 5 weeks	↓ TEWL^∗^, ↓ severity of sodium dodecyl sulfate-induced dermatitis	↑ Keratinocyte differentiation and expression of profilaggrin
Won et al., 2011	NC/Nga mice (AD mouse model)	*L. plantarum* CJLP55, CJLP133 and CJLP136 (1 × 10^10^ CFU/d × 55 days)	Suppression of house-dust mite-induced dermatitis, ↓ epidermal thickening	↑ IL-10 production and alteration of the Th1/Th2 balance
Kim et al., 2012	SKH-1 hairless mice (AD mouse model)	*L. rhamnosus* (Lcr35) (1×10^9^ CFU/d)	↓ TEWL, erythema and inflammation after exposure to topical allergen ovalbumin	↓ IL-4 and TSLP^∗^ via mechanism involving ↑ CD4+CD25+Foxp3+ regulatory T cells
Levkovich et al., 2013	C57BL/6 wild type and IL-10-deficient mice	*L. reuteri* ATCC 6475 (3.5 × 10^5^ organisms/d × 20–24 weeks)	Thicker, shinier fur. ↑ dermal thickness, folliculogenesis, and sebocyte production.	IL-10 dependent anti-inflammatory pathway
Poutahidis et al., 2013	C57BL/6 wild type, oxytocin-deficient WT and KO ^∗^B6; 129S-Oxttm1Wsy/J mice	*L. reuteri* ATCC-PTA-6475 (3.5 × 10^5^ organisms/d in drinking water × 2–3 weeks)	Accelerated wound healing	Oxytocin-mediated regulatory T cell trafficking (↑ Foxp3+ regulatory T cells)
Horii et al., 2014	Wistar rats and hairless Wistar Yagi (HWY) rats	*L. brevis* SBC8803 (0.1 mg/mL in drinking water)	↓ Cutaneous arterial sympathetic nerve activity, ↑ cutaneous blood flow, ↓ TEWL	Activation of 5-HT3 receptors
Kim H.M. et al., 2014	Hairless mice	*L. plantarum* HY7714 (100 μl PBS^∗^/d with 1 × 10^9^ CFU, 1 h prior to UVB irradiation)	↓ Development of wrinkles (number, depth and area) following UVB radiation; ↓ UVB-induced epidermal thickness	Inhibition of MMP-13 expression, MMP-2 activity, and MMP-9 activity in dermal tissue
Lee et al., 2016	NC/Nga mice (AD mouse model)	*L. rhamnosus* IDCC 3201 (1 × 10^8^, 1 × 10^9^, or 1 × 10^10^ cells/d × 8 weeks)	Dose-dependent: ↓ Dermatitis scores, ↓ frequency of scratching, ↓ epidermal thickness	Suppression of mast cell mediated inflammation (↓ mast cells, ↓ IgE, ↓ IL4)
Chen et al., 2017	BALB/c imiquimod-induced psoriasis-like mice	*L. pentosus* GMNL-77 (5 × 10^7^ CFU/0.2 mL/d or 5 × 10^8^ CFU/0.2 mL/d × 7 days)	↓ Erythema, ↓scaling	↓ Expression of pro-inflammatory cytokines (TNF-α, IL-6, and IL-23/IL-17A axis-associated cytokines) mediated by suppression of APCs^∗^(CD103+ dendritic cells) or direct effect on differentiation or proliferation of T cells

*^∗^L. casei, *Lactobacillus casei*, CFU, colony-forming units; DNFB, dinitrofluorobenzene; LCs, Langerhans cells; TEWL, transepidermal water loss; TSLP, thymic stromal lymphopoietin; PBS, phosphate-buffered saline; APCs, antigen-presenting cells*.

**Table 2:** Evidence of beneficial probiotic interventions: human studies.

Author	Study design	Oral probiotic	Clinical response	Proposed mechanism
Siver, 1961	Intervention group only; 300 subjects with acne	*L. acidophilus* and *L. bulgaricus^∗^* (probiotic × 8 days, 2-weeks washout, then re-introduction × 8 days)	clinical improvement in 80% of patients, particularly those with inflammatory acne	Mechanism not established
Peguet-Navarro et al., 2008	Randomized, double-blind, placebo-controlled; 54 healthy subjects	*L. johnsonii* (La1) for 6 weeks	↑ Recovery of skin immune homeostasis following UV-induced immunosuppression	Normalization of epidermal expression of CD1a
Vijayashankar and Raghunath, 2012	Case report; 47-year-old female with severe pustular psoriasis	*L. sporogenes* (Supplementation 3×/d)	Clinical improvement at 15 days, almost complete clearance at 4 weeks	Mechanism not established
Groeger et al., 2013	Randomized, double-blind, placebo-controlled; 26 subjects with plaque psoriasis	*B. infantis ^∗^*35624 (1×10^10^ CFU^∗^/d × 8 weeks)	↓ Systemic inflammation (↓ CRP^∗^, ↓ TNF-α^∗^)	Induction of mucosal immunoregulatory responses that can exert systemic effects
Guéniche et al., 2013	Randomized, double-blind, placebo-controlled; 64 females with sensitive skin	*L. paracasei* NCC2461 (ST11) (1×10^10^ CFU/d × 2 months)	↓ Skin sensitivity, ↓ TEWL^∗^	↓ Skin sensitivity neuromediators and neurogenic inflammation, positive effect on skin barrier function via ↑ circulating TGF-β^∗^
Jung et al., 2013	Randomized, controlled, open-label; 45 females with acne	*L. acidophilus* (NAS), LB-51^∗^, *B. bifidum*, (5×10^9,^ 5×10^9,^ 20×10^9^ CFU 2×/d × 12 weeks	Significant ↓ in number of acne lesions with using probiotic together with Abs than Abs alone	Synergistic anti-inflammatory effect
Lee et al., 2015	Randomized, double-blind, placebo-controlled; 129 females with dry skin and wrinkles	*L. plantarum* HY7714 (1×10^10^ CFU/d × 12 weeks)	↑ Skin hydration, ↓ TEWL, ↑ skin elasticity, ↓ wrinkle depth	Molecular control of signaling pathways and gene expression in skin cells
Fabbrocini et al., 2016	Randomized, double-blind, placebo-controlled; 20 adults with acne	*L. rhamnosus* SP1 (3 × 10^9^ CFU/d (75 mg/d) × 12 weeks)	Improved appearance of adult acne	Normalized skin expression of genes involved in insulin signaling (↓ IGF-1^∗^ expression, ↑ FOXO1)
Ogawa et al., 2016	Randomized, double-blind, placebo-controlled; 126 subjects with elevated TEWL	*L. brevis* SBC8803 (25 or 50 mg/d × 12 weeks)	↓ TEWL, ↑ corneal hydration	Stimulation of serotonin release from intestinal enterochromaffin cells → ↑ vagal nerve activity

*^∗^L. bulgaricus, *Lactobacillus bulgaricus*; *B. infantis, Bifidobacterium infantis;* CFU, colony-forming units; CRP, C-reactive protein; TNF-α, tumor necrosis factor alpha; LB, *Lactobacillus bulgaricus;* TEWL, transepidermal water loss; TGF-β, transforming growth factor beta*.

## Gut Microbiota and Skin Allostasis

The intestinal microbiome contributes to skin allostasis, the restoration of homeostasis after a disturbance or stressor, through gut microbiota-mediated effects on both innate and adaptive immunity (Benyacoub et al., 2014; Kim Y.G. et al., 2014; Chen et al., 2017). Studies have demonstrated that gut bacteria can positively impact the response to disturbed skin barrier function. For example, a study by Baba et al. (2010) demonstrated that the administration of *Lactobacillus helveticus* decreased the severity of sodium dodecyl sulfate-induced dermatitis and subsequent TEWL. Another study showed improved recovery of skin barrier function and decreased signs of reactive skin inflammation – including mast cell degranulation, vasodilation, edema, and tumor necrosis factor alpha (TNF-α) release – following the administration of *Lactobacillus paracasei* CNCM I-2116 (ST11) (Branchet-Gumila et al., 1999; Guéniche et al., 2010; Philippe et al., 2011). Research conducted by Poutahidis et al. (2013) found that mice experienced accelerated wound healing following the consumption of *Lactobacillus reuteri*. Microscopic examination of wounds throughout the healing process revealed the usual histomorphologic stages of wound healing in both probiotic-treated and untreated mice, however, the time required for complete healing was markedly reduced in the treated group. Foxp3+ regulatory T cells were the prominent immune cell population in wound sites among the treated group, while neutrophils were almost completely absent. *L. reuteri*-induced oxytocin-mediated regulatory T cell trafficking resulted in the rapid clearance of neutrophils from the wounds of the treated group, ultimately resulting in decreased time-to-heal (Poutahidis et al., 2013).

The gut microbiome has also been shown to support restoration of skin homeostasis after ultraviolet (UV) radiation exposure. In one study, 10 days of oral supplementation with *Lactobacillus johnsonii* in hairless mice protected the mice against UV-induced contact hypersensitivity, an effect attributed to reduced epidermal Langerhans cells and increased systemic IL-10 levels (Guéniche et al., 2006). In a placebo-controlled study, *Lactobacillus johnsonii* La1 supplementation protected cutaneous immune homeostasis in 54 healthy volunteers following UV radiation exposure. This effect was mediated by the normalization of epidermal expression of CD1a, a transmembrane glycoprotein structurally similar to major histocompatibility complex that presents self and microbial glycolipids to T cells (Dougan et al., 2007; Peguet-Navarro et al., 2008).

Commensal gut flora can promote skin allostasis by influencing T cell differentiation in response to various immune stimuli. Oral administration of *Lactobacillus casei* DN-114 001 has been shown to impair differentiation of CD8+ T cells into cutaneous hypersensitivity effector cells and decrease their recruitment to the skin when exposed to 2-4-dinitrofluorobenzene (**Table 1**). This microbe also increased recruitment of FoxP3+ regulatory T cells to the skin, resulting in decreased apoptosis-mediated skin inflammation, thereby restoring homeostasis through immune-modulatory mechanisms (Chapat et al., 2004; Hacini-Rachinel et al., 2009).

Th17 cells are abundant in both the skin and intestine, as both organs contact the external environment (Weaver et al., 2013). These cells and their pro-inflammatory cytokines are thought to directly contribute to the pathogenesis of several chronic inflammatory dermatoses including psoriasis, Behcet’s disease, and contact hypersensitivity (Van Beelen et al., 2007; Esplugues et al., 2011; Huang et al., 2012). The balance between Th17 effector cells and their counterpart regulatory T cells is greatly influenced by the intestinal microbiome (Van Beelen et al., 2007). Th17 cells can be eliminated in the intestinal lumen, or they may acquire a regulatory phenotype with immunosuppressive characteristics (rTh17) that restricts pathogenicity (Esplugues et al., 2011).

## Dysbiosis and Skin Dyshomeostasis

Intestinal dysbiosis, a state of microbial imbalance, has the potential to negatively impact skin function. Free phenol and p-cresol, metabolic products of aromatic amino acids, are considered biomarkers of a disturbed gut milieu as their production is induced by certain pathogenic bacteria, most notably *Clostridium difficile*. These metabolites can access the circulation, preferentially accumulate in the skin, and impair epidermal differentiation and skin barrier integrity (O’Neill et al., 2016). Indeed, high p-cresol serum levels are associated with reduced skin hydration and impaired keratinization (Dawson et al., 2011; Miyazaki et al., 2014). Intestinal dysbiosis results in increased epithelial permeability which then triggers the activation of effector T cells, disrupting their balance with immunosuppressive regulatory T cells. Pro-inflammatory cytokines further enhance epithelial permeability and set up a vicious cycle of chronic systemic inflammation (Kosiewicz et al., 2014; O’Neill et al., 2016). These are just a few mechanisms by which a disturbed gut microbiome manifests in impaired skin function. Here, we will discuss mechanisms by which intestinal dysbiosis contributes to three common skin disorders – acne, AD, and psoriasis.

### Acne Vulgaris

Acne vulgaris is a chronic disease of the pilosebaceous unit that manifests clinically as non-inflammatory comedones or inflammatory papules, pustules, and nodules (Yentzer et al., 2010; Bhate and Williams, 2013). Three primary factors are implicated in its pathophysiology – sebum oversecretion, abnormal keratinocyte desquamation leading to ductal obstruction, and superimposed inflammation mediated by *Propionibacterium acnes* (Dawson and Dellavalle, 2013; Agak et al., 2014; Fox et al., 2016; Dreno et al., 2017; Picardo et al., 2017; Rodan et al., 2017).

Approximately 85% of adolescents and young adults between the ages of 12 and 25 are affected by acne, and it represents the eighth most common medical disorder worldwide (Hay et al., 2014; Tan and Bhate, 2015; Lynn et al., 2016; Zaenglein et al., 2016). Acne is particularly prevalent in western countries, a phenomenon thought to be related to an abundance of carbohydrates in the typical western diet. A high glycemic load promotes an increase in insulin/insulin-like growth factor (IGF-1) signaling. This is thought to induce increased cytoplasmic expression of the metabolic forkhead box transcription factor (FoxO1), a sensor of cell nutrition state. FoxO1 ultimately triggers mammalian target of rapamycin complex 1 (mTORC1), a governor of metabolism and cell proliferation, to mediate sebaceous gland hyperproliferation, lipogenesis, and hyperplasia of acroinfundibular keratinocytes, thereby contributing to the development of acne (Melnik, 2015; Agamia et al., 2016; Zaenglein et al., 2016).

Gut microbiota influence the pathophysiology of acne via cross talk between intestinal commensal bacteria and the mTOR pathway (Noureldein and Eid, 2018). Metabolites produced by gut microbiota have been shown to regulate cell proliferation, lipid metabolism, and other metabolic functions mediated by the mTOR pathway. The mTOR pathway can in turn affect the composition of intestinal microbiota through regulation of the intestinal barrier. In cases of intestinal dysbiosis and disrupted gut barrier integrity, this bidirectional relationship can result in a positive feedback cycle of metabolic inflammation. Given the important role of mTORC1 in the pathogenesis of acne, this relationship serves as a mechanism by which the gut microbiome can influence acne pathophysiology.

The complex connection between acne and GI dysfunction may also be mediated by the brain, an idea first postulated by Stokes and Pillsbury (1930). Supporting this hypothesis is the frequent association of both psychological comorbidities – anxiety and depression – and GI distress with acne. These psychological stressors are hypothesized to cause the intestinal flora to either produce different neurotransmitters – serotonin, norepinephrine and acetylcholine – or trigger nearby enteroendocrine cells to release neuropeptides. These neurotransmitters not only increase intestinal permeability, leading to both intestinal and systemic inflammation, but also directly access the circulation through the compromised intestinal barrier resulting in systemic effects (Zhang et al., 2008; Do et al., 2009; Bowe and Logan, 2011; Bowe et al., 2012, 2014; Zouboulis, 2014; Duman et al., 2016; Jena and Sahoo, 2016; Prakash et al., 2016; Ramrakha et al., 2016; Vaughn et al., 2017). The gut-brain-skin axis hypothesis remained dormant for several decades but has been validated by recent advances in microbiome research and our understanding of its effect on health and disease (Bowe and Logan, 2011; Bowe et al., 2014). Consistent with this hypothesis, an upregulation of substance P containing nerves and a strong expression of this neuropeptide is mutually seen in both acne vulgaris and intestinal dysbiosis. Substance P can trigger inflammatory signals that result in the increase of pro-inflammatory mediators implicated in the pathogenesis of acne (IL-1, IL-6, TNF-α, PPAR-γ) (Lee et al., 2008; Arck et al., 2010; Bercik and Collins, 2014; Borovaya et al., 2014; Theodorou et al., 2014; Petra et al., 2015; Rokowska-Waluch et al., 2016).

Alternatively, the link may originate with GI dysfunction which then leads to psychological and cutaneous disorders. Hypochlorhydria is frequently associated with acne. Low levels of acidity allows for the migration of colonic bacteria to distal parts of the small intestine, creating a state of intestinal dysbiosis and small intestinal bacterial overgrowth (SIBO). A larger bacterial population competes for nutrients and impairs the absorption of fats, proteins, carbohydrates, and vitamins. Malabsorbed nutrients, including folic acid, zinc, chromium, selenium, and *ω*-3 fatty acids have been shown to influence one’s psychological state and, along with systemic oxidative stress, have been implicated in the pathophysiology of acne vulgaris (Katzman and Logan, 2007). SIBO also results in the production of toxic metabolites, which can injure enterocytes, increase intestinal permeability, and ultimately lead to systemic inflammation (Bures et al., 2010; Bowe and Logan, 2011; Bowe et al., 2014).

### Atopic Dermatitis

Atopic dermatitis is the most common chronic pruritic inflammatory dermatosis, affecting 15–30% of children and 2–10% of adults (Simpson et al., 2014; Seite and Bieber, 2015; Bin and Leung, 2016). It is a heterogeneous disorder with a variety of endotypes and phenotypes illustrated by wide variations in clinical features with respect to age, severity, and allergen response (Seite and Bieber, 2015; Bin and Leung, 2016; Irvine et al., 2016; Muraro et al., 2016; Huang et al., 2017).

Over the last few decades, our understanding of the pathogenesis of AD has improved with the discovery of more innate and adaptive immune cells and cytokines (Otsuka et al., 2017). Skin barrier dysfunction and altered immune responses are primary players in the pathogenesis of AD (Seite and Bieber, 2015; Bin and Leung, 2016; Irvine et al., 2016; Muraro et al., 2016; Huang et al., 2017). A compromised barrier secondary to environmental or genetic causes is typically the preceding event in AD development. The principal inherited cause of barrier dysfunction is loss of function mutations in the gene encoding FLG. FLG plays an essential role in maintaining epidermal homeostasis by assisting with water retention and barrier function. Therefore, a mutation in FLG results in increased TEWL as well as increased susceptibility to invasion by environmental antigens (Kelleher et al., 2015; Horimukai et al., 2016).

In the acute stage, allergens breaching a dysfunctional skin barrier will trigger the release of keratinocyte-derived cytokines such as TSLP, IL-33 and IL-25. IL-25 and IL-33 will in-turn activate type 2 innate lymphoid cells through their interaction with IL-17B and IL-1RL1, respectively, resulting in the production of IL-13 and IL-5 which will then stimulate a Th2 immune response. Activation of Th2 cells is further enhanced by TSLP-mediated maturation of Langerhans cells and CD11^+^ dendritic cells. In the chronic stage, IL-22 released from Th22 CELLS promotes the epidermal production of anti-microbial peptides, including defensins, which can participate in skewing the immune response toward a more predominant Th1 response. Tissue remodeling seen in this stage could be secondary to an IL-17 mediated release of pro-fibrotic cytokines such as IL-11 and TGF-β from eosinophils (Otsuka et al., 2017).

Allergic diseases including asthma, hay fever, and eczema have increased in prevalence over the past several decades (Wesemann and Nagler, 2016; Johnson and Ownby, 2017). As developed countries known for their sterile environments saw the most dramatic rise in AD and other allergic diseases, the hygiene hypothesis, first proposed by Strachan (1989), was a widely accepted theory used to explain this phenomenon. Dr. Strachan proposed that allergic disorders arise when our immune system inappropriately responds to harmless antigens via Th2-mediated responses. According to his hypothesis, early exposure to microbial antigens is essential to immune development, as it encourages Th1 rather than Th2-mediated immune responses. With more sterile environments in developed countries, the hygiene hypothesis explains the disproportionate rise of allergic disease in the western world. This theory, however, is flawed in that it fails to explain the parallel increase in prevalence of Th1-mediated autoimmune diseases. Another proposed explanation, the diet-microbiome theory, seeks to reconcile this flaw.

The diet-microbiome theory implies that the increased prevalence of allergic disease stems from a less robust state of immune homeostasis rather than from overresponse to innocuous environmental cues (Kim et al., 2013; Purchiaroni et al., 2013; Song et al., 2016; Johnson and Ownby, 2017). According to this theory, the gut microbiome’s contribution to immune homeostasis is impaired by the typical western diet. Immune homeostasis begins to take shape early in life through exposure to maternal microbiota, and the infant’s intestinal flora is further developed with exposure to breast milk, other food, and environmental microbes (Sironi and Clerici, 2010; Neu and Rushing, 2011; Clemente et al., 2012; Frei et al., 2012; Rook, 2012; Bendiks and Kopp, 2013; Kim et al., 2013; Purchiaroni et al., 2013; Bloomfield et al., 2016; Song et al., 2016; Johnson and Ownby, 2017). The low fiber and high fat content characteristic of the western diet fundamentally changes the gut microbiome, resulting in deficient production of immunomodulatory metabolites, particularly SCFAs. SCFAs are known for their anti-inflammatory actions mediated by G-protein coupled receptor 43 and for their contribution to epithelial barrier integrity (Maslowski et al., 2009). Anti-inflammatory activity is further mediated by regulatory T cells and driven by TGF-β and/or interleukin 10 (IL-10). IL-10 exerts its inhibitory function by inducing TGF-β and other cytokines as well as suppressive signaling molecules including CTLA-4 and PD-1. Reduced local and systemic immune tolerance resulting from an altered gut microbiome may help explain the observed rise of both autoimmune and atopic disease observed in the western world (Maslowski et al., 2009; Agrawal et al., 2011; Purchiaroni et al., 2013; Seite and Bieber, 2015; Johnson and Ownby, 2017).

Studies have sought to demonstrate the link between intestinal dysbiosis and atopic disease (Johnson and Ownby, 2017). In two Korean studies, metagenomic analysis of fecal samples from patients with AD demonstrated a significant reduction in *Faecalibacterium prausnitzii* species compared to control patients. A parallel decrease in SCFA production among AD patients was observed as well. The authors emphasized a possible positive feedback loop between intestinal dysbiosis regarding *F. prausnitzii* and epithelial barrier disruption secondary to uncontrolled epithelial inflammation (Kim et al., 2013; Song et al., 2016). Disruption in the intestinal barrier contributes to this feedback loop by allowing the penetration of poorly digested food, microbes, and toxins into the circulation to reach target tissue, including the skin, where they trigger Th2 immune responses resulting in further tissue damage (Purchiaroni et al., 2013; Seite and Bieber, 2015; Song et al., 2016; Johnson and Ownby, 2017).

### Psoriasis

Psoriasis is an immune-mediated chronic relapsing-remitting inflammatory dermatosis triggered by a multitude of environmental and internal factors in genetically susceptible individuals (Parisi et al., 2013; Rachakonda et al., 2014; Kulig et al., 2016; Takeshita et al., 2017). Histologic features include acanthosis, reflective of a state of keratinocyte hyperproliferation, and parakeratosis, indicative of dysregulated keratinocyte differentiation. Increased vascularity is characteristic as well, allowing for the accumulation of inflammatory subpopulations of neutrophils, dendritic cells, and T lymphocytes (Roberson and Bowcock, 2010; Kulig et al., 2016). Clinically, psoriasis commonly presents as recurrent episodes of well-demarcated scaly erythematous plaques but can rarely also manifest as generalized life-threatening erythroderma (Mari et al., 2017).

Treatment options have evolved as the pathophysiology of psoriasis has become better understood. Initially considered merely a hyperproliferative skin disorder, treatment once focused on anti-proliferative therapeutic modalities. In the 1980s, the Th1 subset of effector T cells their derived cytokines became the target of many psoriasis therapeutics. More recently, after the discovery of elevated IL-17 levels in psoriatic lesions, therapies have focused on Th17 cells, a novel subset of T cells, as a principal player. Th17 cytokines enhance the expression of the IL-10 cytokine family including; IL-20 and IL-22 cytokines capable of promoting the hyperproliferation of keratinocytes. Following the discovery of the Th17 pathway, most clinical and mechanistic evidence suggests that psoriasis is primarily driven by the IL-23/IL-17/Th17 axis (Baba et al., 2006; Fitch et al., 2007; Guttman-Yassky et al., 2008; Ma et al., 2008; Gaffen et al., 2015; Coates et al., 2016).

Psoriasis is commonly accompanied by inflammation in other organ systems. Seven to 11% of inflammatory bowel disease (IBD) patients are diagnosed with psoriasis, making the association with GI inflammation particularly strong (Huang et al., 2012; Eppinga et al., 2014; Takeshita et al., 2017). Certain shared genetic and environmental factors as well as immune pathways have been implicated in the etiopathogenesis of both diseases (Huang et al., 2012). For example, Th17 cells and their cytokines, known to play a principal role in the development of psoriasis, have been implicated in the pathophysiology of IBD as well (Eppinga et al., 2014; Verstockt et al., 2016). This subset of cells is also thought to play a role in the development of ankylosing spondylitis and rheumatoid arthritis, two autoimmune inflammatory joint diseases commonly reported in patients with psoriasis and IBD (Zhang et al., 2012). Evidence such as this suggests another possible tri-directional axis orchestrated by the gut and its influence on the skin (Eppinga et al., 2014).

Metabolites produced by the intestinal microbiome have immune-modifying potential, capable of altering the balance between immune tolerance and inflammation through their influence on the differentiation of naïve T cells into either regulatory or Th17 lineages. Effector T cells are generally anabolic and depend on glycolysis as their source of adenosine triphosphate (ATP). Memory and resting T cells, however, are considered catabolic and utilize fatty acids and amino acids, in addition to glucose, to generate ATP through oxidative phosphorylation. The primary transcription factors of the lipogenic and glycolytic pathways are adenosine monophosphate activated kinase and rapamycin, respectively. Both serve as energy sensors and are regulated by the accessibility of nutrients in the gut milieu, which can be modulated by gut microbiota (Omenetti and Pizarro, 2015).

The pattern of dysbiosis found in IBD patients has also been described in psoriatic patients with and without IBD (Scher et al., 2015). There is depletion of symbiont bacteria, including *Bifidobacteria*, *Lactobacilli*, and *Faecalibacterium prausnitzii* as well as colonization with certain pathobionts such as *Salmonella, Escherichia coli, Helicobacter, Campylobacter, Mycobacterium*, and *Alcaligenes.* One study revealed a decreased presence of *Parabacteroides* and *Coprobacillus*, two beneficial gut species, in psoriasis and psoriatic arthritis patients comparable to what is observed in patients with IBD. Reduced presence of beneficial phyla may translate into functional consequences including poor regulation of intestinal immune responses that may then affect distant organ systems (Scher et al., 2015). *F. prausnitzii*, one of the most common microbial inhabitants of the large intestine, provides many benefits to the host. It serves as an important source of butyrate, a SCFA that provides energy for colonocytes, reduces oxidative stress, and imparts anti-inflammatory action by triggering regulatory T cells, thereby conferring immune tolerance that extends beyond the GI system (Sokol et al., 2008; Lopez-Siles et al., 2012). Psoriatic patients harbor a significantly lower number of this microbe compared to healthy controls (Eppinga et al., 2016). It has also been theorized that the far-reaching effects of intestinal dysbiosis are the result of gut microbes and their metabolites breaching an impaired intestinal barrier and entering systemic circulation to directly target distant organs, including the skin and joints. Consistent with this hypothesis, DNA of gut microbial origin has been isolated from the blood of patients with active psoriasis (Ramírez-Boscá et al., 2015).

## Modulation of the Gut Microbiota for Treatment and Prevention (Rehabilitation of the Gut Ecosystem)

The gut microbiome is greatly influenced by diet. Though long-term dietary habits shape bacterial composition, dramatic modulation of diet over the short term can rapidly alter gut bacteria as well. Given the gut microbiome’s influence on inflammatory disease, this provides an opportunity to intentionally modify the microbiome with therapeutic aims (Huang et al., 2017). Probiotic supplementation, the administration of live beneficial gut bacteria, has a promising potential role in the prevention and management of various skin conditions (Krutmann, 2009; Hill et al., 2014; Farris, 2016; Grant and Baker, 2017; Sánchez et al., 2017; Sarao and Arora, 2017). Prebiotics, non-viable bacterial components and metabolites, and synbiotics, the combination of pro- and prebiotics, offer similar health benefits (Muizzuddin et al., 2012; Frei et al., 2015).

### Cosmetic Effect of Probiotics on Skin

Ultraviolet radiation is the primary external contributor to skin aging. Through its stimulation of signaling pathways which ultimately increase the transcription of target genes key to photoaging, UV radiation results in increased laxity, dryness, and pigmentation (Chen et al., 2015; Xia et al., 2015; Wiegand et al., 2016). Activator protein 1 is a matrix metalloproteinase (MMP) transcription factor with a primary role in UVB-induced skin aging. MMPs are zinc-dependent endopeptidases capable of degrading extracellular matrix macromolecules. Interstitial collagenase (MMP-1) cleaves collagen I and III fibrils in skin, while MMP-9 further degrades these fibrils into smaller peptides (Quan et al., 2013; Cavinato and Jansen-Dürr, 2017; Qin et al., 2017). MMP-2 and MMP-9, primarily expressed in the epidermis, break down collagen IV and VII found in the epidermal basement membrane (Pittayapruek et al., 2016). When keratinocytes are exposed to UVA radiation, there is increased expression of TNF-α, a pro-inflammatory cytokine (Jeong et al., 2016).

Studies have sought to demonstrate how modulation of the gut microbiome can influence immune signaling pathways in a way that counteracts UV damage. Lipoteichoic acid (LTA), a cell wall component of *Lactobacillus* species, is known for its anti-inflammatory properties (Jeong et al., 2016). In one Korean study, oral administration of *Lactobacillus plantarum* HY7714, resulted in the prevention of UV-induced photoaging in mice through the inhibition of MMP-1 expression in dermal fibroblasts (Kim H.M. et al., 2014). This anti-aging effect was reproduced in human research. In a double-blind, placebo-controlled study, oral supplementation of *L. plantarum* HY7714 in 110 middle-aged Korean subjects for 12 weeks resulted in improved cutaneous elasticity and increased skin hydration (Lee et al., 2015). Another study demonstrated that *Lactobacillus sakei* LTA is capable of reversing UV-induced skin aging through its immune modulating effect on monocytes (You et al., 2013).

### Probiotics and Acne Vulgaris

Topical and oral antibiotics are often included in traditional acne treatment regimens. Though effective, this approach risks antibiotic resistance and disruption of the microbiome. Given the role of intestinal dysbiosis in inflammatory skin conditions, probiotic supplementation represents a promising alternate, or adjuvant, acne treatment approach.

In an early study of probiotic supplementation, the ingestion of *Lactobacillus acidophilus* and *Lactobacillus bulgaricus* probiotic tablets by 300 acne patients resulted in acne improvement in 80% of subjects, particularly in subjects with inflammatory lesions (Siver, 1961; Bowe and Logan, 2011; Bowe et al., 2014). Probiotics can suppress *Propionibacterium acnes* through the secretion of antibacterial protein. *Streptococcus salivarius* and *Lactococcus* HY449 produce bacteriocin-like inhibitory substance and bacteriocins, respectively, which inhibit the growth of *P. acnes* (Bowe and Logan, 2011; Bowe et al., 2014; Kober and Bowe, 2015). In another clinical study, subjects who received oral *Lactobacillus* and *Bifidobacterium* species in conjunction with oral antibiotics experienced a significantly greater decrease in acne lesion count compared to an antibiotic-only control group (**Table 2**) (Volkova et al., 2001; Jung et al., 2013).

In addition to their antimicrobial effects, probiotics can disrupt the pathogenesis of acne through immunomodulatory and anti-inflammatory actions. *In vitro* studies of *Streptococcus salivarius*, a commensal microbe, attributed the anti-inflammatory effect of this strain to inhibition of IL-8 secretion, suppression of the NF-κB pathway, and downregulation of genes associated with the adhesion of bacteria to epidermal surfaces (Cosseau et al., 2008).

Probiotics may also lower the glycemic load, reduce IGF-1 signaling, and ultimately decrease keratinocyte proliferation and sebaceous gland hyperplasia. In one pilot study, the consumption of *Lactobacillus rhamnosus* SP1 for 12 weeks resulted in reduced expression of IGF-1 and oxidative stress markers (**Table 2**) (Fabbrocini et al., 2016). In a study of obese diabetic mice, administration of *Bifidobacterium* species resulted in inhibition of high-fat diet-induced endotoxemia and inflammation via a GLP-2-dependant mechanism. Increased glucagon-like peptide 2 (GLP-2), an intestinotrophic proglucagon-derived peptide, resulted in improved tight junction integrity and reduced intestinal permeability (Cani et al., 2009).

### Probiotics and Atopic Dermatitis

The primary treatment approach to AD combines topical emollients and anti-inflammatory drugs to compensate for disrupted barrier function and poor immune tolerance, respectively (Muraro et al., 2016). Given the gut microbiome’s integral role in immune development and homeostasis, probiotics may be useful in both the prevention and treatment of allergic disorders including AD via microbial, epithelial, and immune effects. Probiotics modify microbial composition, prevent pathogen invasion by competitively binding to epithelial cells, and suppress growth of pathogens by secreting bacteriocin. They also contribute to the restoration of impaired barrier function by increasing the expression of tight junction proteins as well as the production of SCFAs. Immune benefits include the inhibition of proinflammatory cytokines (IL-4, INFγ, IL-17) and the promotion of anti-inflammatory cytokines (IL-10, TGF-β). Probiotics can increase the number of regulatory T cells that suppress the cutaneous expression of thymic stromal lymphopoietin involved in the stimulation of dendritic cells, effectively preventing the differentiation of naïve T cells into Th2 and Th17 subtypes. Regulatory T cells can migrate to the skin and inhibit Th2 and Th17 responses, thereby exerting a therapeutic role in addition to a preventative one (Kim et al., 2013; Chang et al., 2016; McCusker and Sidbury, 2016; Rather et al., 2016).

*Lactobacillus* and *Bifidobacterium* species are the most commonly tested probiotics in AD (Kim et al., 2010; Enomoto et al., 2014). Oral supplementation with *Lactobacillus rhamnosus* Lcr35 in an AD mouse model resulted in the upregulation of CD4+CD25+Foxp3+ regulatory T cells and the downregulation of interleukin-4 and thymic stromal lymphopoietin (**Table 1**) (Kim et al., 2012). In a separate study, supplementation of another AD mouse model with *Lactobacillus plantarum* CJLP55, CJLP133, and CJLP136 resulted in inhibition of house dust mite-induced dermatitis via increased production of IL-10 and alteration of the Th1/Th2 balance (Won et al., 2011). After supplementation with *Lactobacillus rhamnosus* IDCC 3201, there was suppression of mast cell mediated inflammation in the same mouse model (Lee et al., 2016).

In humans, prenatal and postnatal probiotics have proven efficacy in the management, and even prevention, of AD in high risk infants. In one placebo-controlled study, *Bifidobacterium bifidum* BGN4, *Bifidobacterium lactis* AD011, and *Lactobacillus acidophilus* AD031 supplements were given to pregnant Korean women with a positive family history of AD 4–8 weeks before delivery and to their infants for the first 2 months of life. The incidence of AD was significantly lower in the probiotic-treated group compared to the control group (Kim et al., 2010). In a separate study, prenatal and postnatal probiotic milk supplementation in Norwegian women and their infants was also associated with a reduced incidence of AD (Bertelsen et al., 2014). In a third study, maternal and infant supplementation with *Bifidobacterium breve* M-16V and *Bifidobacterium longum* BB536 further supported the preventative effect of probiotics in AD (Enomoto et al., 2014). Regarding AD treatment, a recent meta-analysis was conducted of studies investigating the efficacy of probiotics in AD symptom-control. This analysis concluded that probiotic use improved AD, reflected by a significant reduction in the Severity Scoring of AD index, in all ages except infants under 1 year (Chang et al., 2016).

### Probiotics and Psoriasis

Data on probiotic supplementation in psoriasis treatment are limited, but promising outcomes have been documented. One study evaluating the effect of *Lactobacillus pentosus* GMNL-77 on an imiquimod-induced psoriasis mouse model found that probiotic-treated mice experienced significantly less erythema, scaling, and epidermal thickening compared to untreated control mice (Chen et al., 2017). Oral administration of *L. pentosus* GMNL-77 appeared to suppress expression of TNF-α, IL-6, and proinflammatory cytokines in the IL-23/IL-17 cytokine axis. Though the mechanism for reduced T cell activity was unclear, the study authors proposed that this effect was mediated by suppression of CD103+ dendritic cells, intestinal antigen presenting cells that have been shown to modulate regulatory T cells in the GI tract. In a separate placebo-controlled study of psoriasis patients, *Bifidobacterium infantis* 35624 supplementation led to significantly reduced plasma levels of TNF-α in the probiotic-treated group (**Table 2**) (Groeger et al., 2013). In one documented case of severe pustular psoriasis unresponsive to steroids, dapsone, and methotrexate, clinical improvement was observed within 2 weeks of initiating *Lactobacillus sporogenes* supplementation three times per day, with almost complete resolution achieved at 4 weeks (Vijayashankar and Raghunath, 2012).

## Conclusion and Future Perspectives

Basic science research and clinical studies have demonstrated the gut microbiome’s contribution to host homeostasis, allostasis, and the pathogenesis of disease. Through complex immune mechanisms, the influence of the gut microbiome extends to involve distant organ systems including the skin. With intentional modulation of the microbiome, probiotics, prebiotics, and synbiotics have proven beneficial in the prevention and/or treatment of inflammatory skin diseases including acne vulgaris, AD, and psoriasis. In this up-and-coming field, future research should improve our understanding of the complex mechanisms underlying the gut-skin axis, investigate the therapeutic potential of long-term modulation of the gut microbiome, and potentially expand therapeutic manipulation to include commensal gut fungi and viruses in order to fully harness the gut microbiome’s influence in the treatment of skin disease.

## Author Contributions

IS and AR were responsible for data research and collation; NI for manuscript review, and MG for overall review.

## Conflict of Interest Statement

The authors declare that the research was conducted in the absence of any commercial or financial relationships that could be construed as a potential conflict of interest.
